# Extending the notion of customer value to surfing camps

**DOI:** 10.1016/j.heliyon.2021.e07876

**Published:** 2021-08-26

**Authors:** Brad Wilson, Paulo Rita, Andres Barrios, Bastian Popp

**Affiliations:** a Universidad de Los Andes, Bogotá, Colombia; b NOVA Information Management School (NOVA IMS), Universidade NOVA de Lisboa, Portugal; c Universität des Saarlandes, Germany

**Keywords:** Customer value, Service-dominant logic, Surf camps, Tourism marketing

## Abstract

This study applies the germane principles of service-dominant logic by investigating how different dimensions of service value impact customers’ satisfaction and related behavioral intentions in the surf camps context. An empirical model was developed and tested via survey responses from 300 Portuguese surf camps tourists who profiled their experience. Data were analyzed using Structural Equation Modeling, specifically Partial Least Squares (PLS-PM). Results highlight the respective impact the numerous value dimensions (functional, emotional, social, epistemic, experiential, and contextual) have on the overall level of perceived value as well as its resultant impact on satisfaction and repurchase intentions.

## 1 Introduction

Surfing has gone mainstream (Martin & Assenov, 2014; Towner and Milne, 2017) with many people wanting a surfing experience, and companies exploiting it commercially via products (e.g., Quicksilver and Ripcurl clothing lines) or services (e.g., sport events). There are currently many surfing-related companies, schools, shops, equipment, and events. An important surfing-related business is tourism, which involves thousands of accommodation units (resorts and homestays), channel distributors (tour operators, wholesalers, and travel agents) as well as vertically integrated services (Barbieri and Sotomayor, 2013; Ratten, 2018). Although there is no commonly accepted official definition for surf tourism (Barbieri and Sotomayor, 2013; Porter and Usher, 2019), Fluker (2003, p.7) notes some elements of this activity as follows:

“The act of people travelling to either domestic locations for a period of time not exceeding 6 months, or international locations for a period of time not exceeding 12 months, who stay at least one night, and where the active participation in the sport of surfing, where the surfer relies on the power of the wave for forward momentum, is the primary motivation for selection.”

Surf tourism can be divided into two different components: (1) recreational surf travel, that is, surfers planning their own trips, using their own transport and equipment, and staying in local accommodation, campervans or in their own tents; (2) commercial surf tourism, that is, surfers using a planned tourism package with all the logistic aspects of the trip organized, generally including transport, accommodation, equipment, and food where surfers intend to devote their active leisure time to surfing (Buckley, 2002).

Surf camps, one of the most popular formats of commercial surf tourism, are part of the specialized services offered in surf recreation and arose from the surfers’ need for accommodation while traveling around the coastline. This accommodation is mostly located in villages, cities, or regions that are popular for their surf breaks often perceived as surf tourist spaces. Surf camps are characterized by small beachfront hotels or guesthouses that fulfill the needs and wants of national and international surfers including services like accommodation, food, surf instruction, and guided trips, with their offer varying from basic camps to luxury facilities for both novice and experienced surfers.

On the demand side, surf camp tourists include a diversified profile of surfers: from first time surfers to beginners, intermediate, advanced, or professional standard levels. The primary motivations of these tourists can include the need of either getting “perfect waves” or just trying surfing as part of their coastal holiday. The surfers usually select a set of desired destinations after recognizing their needs. Surfers and non-surfers learn and research the latest spots and camps through films, TV, literature, magazines, CDs, and DVDs (Ponting and McDonald, 2013). Despite recognizing that the factors the surf tourists consider when selecting surf camps are likely to be similar to those found in regular tourism, it is important to understand that the overall surfing appeal is the most relevant factor when selecting a destination (Barbieri and Sotomayor, 2013).

While different studies have analyzed the experience of surfing and the hospitality industry that surrounds it (e.g., Brochado et al., 2018), appropriate ways to measure the value provided by these settings still need to be investigated. Therefore, the aim of this research is to assess the value surfers derive from surf camps. In particular, our study integrates the Service-Dominant Logic (SDL) notion (Vargo & Lusch, 2004, 2008, 2016) that provides a more holistic summation of the assessment of the entire hospitality service experience. This study utilizes the Customer Perceived Value (PERVAL) framework (Sweeney and Soutar, 2001) to analyze the value configuration service space of surf camp lodgings, including the level of tourism value on participants of surf camps in Portugal, and its influence on consumers’ overall satisfaction level and future intentions to recommend or revisit.

The paper is organized as follows. First, we develop a review of the marketing notions of value, customer value, satisfaction, and behavioral intentions (i.e., loyalty), and present a novel structural empirical model incorporating these constructs. Then, we describe the study's empirical setting of surf camps in Portugal, the data collection process, and the planned analytical approach. The empirical findings are discussed in relation to their implications for surf camps and the hospitality industry. Finally, we discuss how the SDL view provides guidance to tourism entrepreneurs on how to develop value co-creation experiences that increase consumer satisfaction and positively impacts loyalty levels.

## 2 Literature review

### 2.1 Marketing notion of value

The notion of value has achieved key role status within marketing. Traditionally, value has been understood as firm-centric and referred to as the chief outcome of an organization's product, being equated to utility (Hwang and Lyu, 2018; Sánchez-Fernández and Iniesta-Bonillo, 2007; Zeithaml et al*.*, 2020). Value is created when a firm provides a product that satisfies a consumer who pays a price to the firm. Here, value is a unidimensional construct resulting from the trade-off between the customer's satisfaction with a product and the respective price paid (Gallarza and Saura, 2006; Gallarza and Gil, 2008).

New perspectives of value have emerged, such as the view suggested by SDL (Vargo & Lusch, 2004, 2008, 2017) that considers its interactive, contextual, and dynamic nature in the marketplace. According to SDL, value emerges from a collaborative process between the organization and its customers (Vargo and Lusch, 2008). Using a mathematical analogy, the SDL intimates that during a service encounter, the actors (organization and its customers) use their *operant resources* defined as those that enable actions (e.g., knowledge, skills) to integrate their *operand resources defined as those that are acted upon* (e.g., natural environment, infrastructure) to co-create value (Vargo and Lusch, 2004). Such value co-creation takes place under unique usage situations and is always determined by the beneficiaries depending on their context (Vargo and Lusch, 2008; Chandler and Vargo, 2011).

The multidimensional notion of value proposed by SDL can be considered more appropriate to evaluate how value is co-created in the hospitality service context, as these approaches are better capable of dealing with the heterogeneous and continued interaction within and between producers and consumers (Williams and Soutar, 2009). In addition, the particularities of SDL characteristics constitute a suitable framework for surf camps where the eventual goods involved such as surrounding landscapes, port, and accommodation facilities, interplay with surfers' knowledge and skills for enhancing their experiences. Next, with SDL as the foundation we present literature pertaining to customers’ perceived value (PV) assessment while considering the impact value may have on customer satisfaction (CS) and behavioral intentions (BI). A conceptual model is proposed, which forms the basis for testing.

### 2.2 Customer perceived value, satisfaction, and behavioral intentions

PV has been addressed following three research streams: (1) price-based studies on the classification and analysis of the quality-price relationship (Monroe and Chapman, 1987) whereby the empirical operationalization of the construct qualifies this element as antecedents, instead of summated components of value; (2) means-end theory (used by Zeithaml, 1988) defining PV as a bi-directional trade-off between what is sacrificed versus what is received in an exchange; (3) emphasizing the hedonic component of consumption (Babin and Darden, 1994; Bleier et al., 2019; Dedeoğlu, 2019), reflecting entertainment and emotional worth of shopping, non-instrumental, experiential and affective, going beyond a utilitarian value perspective of value (i.e., instrumental, task-related, rational, functional, cognitive, and a means to an end).

From a marketing perspective, CS is an emotional feeling developed in response to confirmation/disconfirmation of value perceptions (Williams and Soutar, 2009; Pereira et al., 2016, 2017; Zhao et al., 2019). PV interrelates with attributes/dimensions forming an impression of phenomenon, reflecting the complexity of consumers' perceptions of value (Sánchez-Fernández and Iniesta-Bonilo, 2007). When starting the buying process, individuals tend to frame their own perceptions about how a product/service is likely to perform in the future (del Bosque and San Martín, 2008). Perceptions are usually an evaluative judgment, whereas the term value refers to the standards, rules, criteria, norms, goals, or ideals that serve as the basis for such an evaluative judgment. A performance that exceeds expectations results in a positive disconfirmation. In the reverse scenario, negative disconfirmation occurs. Hence, PV is a critical element for consumers’ consumption and in the decision-making process (Williams and Soutar, 2009).

PV occurs at various stages of the purchase process while CS is a post-purchase evaluation (Sweeney and Soutar, 2001). Cronin et al. (2000) identified a positive relationship between PV and CS. There also appears to be a strong link between CS and repurchase intentions, which shows a positive correlation between customers’ perceptions of service quality and their repurchase intentions (Williams and Soutar, 2009). Consequently, PV is not only influential at the final purchase phase but also impacts CS, intention to recommend, and repurchase behavior in the post-purchase phase (Al-Sabbahy et al., 2004). Different areas of consumer behavior such as product choice, satisfaction, and repeat purchasing can be explained through value constructs (Gallarza and Saura, 2006), with higher satisfaction levels often leading to higher levels of loyalty. Furthermore, nurturing PV is considered to be one of the most important dimensions for bolstering competitive advantage and suitable business strategies need to be identified to achieve marketplace advantages (Sweeney and Soutar, 2001).

### 2.3 Perceived value in the tourism context - PERVAL

The SDL perspective highlights value as a dynamic and multidimensional construct consisting of several interrelated elements, which can be notably examined through the lenses of human perceptions and experiences (Vargo and Lusch, 2008). One multidimensional framework of value in services that has the potential to meet this claim and has been successfully applied to tourism is PERVAL (Sweeney and Soutar, 2001). The PERVAL framework allows measuring PV from a utilitarian value component (functional value and value for money) and from a contextual view including socio-psychological dimensions (social value, epistemic value, and emotional value). The framework underscores the notion that consumers assess services not just in functional terms of expected performance and value for money, but also in terms of the enjoyment or pleasure derived from the product (emotional value), the social consequences of what the product communicates to others (social value), and the novelty dimension (i.e., escaping from the daily routine) that is usually reflected in a tourism context (epistemic value).

Although the PERVAL scale represents an important measurement advancement of PV in the service context (Boksberger and Melsen, 2011), it needs to be adapted to be suitable for the tourism context to capture context-specific value dimensions. This paper therefore extends this model by adding two new components related to surf camps addressing information specific to their experiential and contextual elements.

### 2.4 Conceptual model and hypotheses development

The following model integrates the SDL notions of value with customers’ value perception and links their satisfaction level with behavioral intentions to repurchase and consequently recommend. PV consists of formative components to identify the different value dimensions and their subsequent impact on satisfaction, which also serves as a determinant construct, influencing loyalty and willingness to recommend (Williams and Soutar, 2009).

The model starts with the PERVAL framework, which represents an important advancement in the measurement of PV in the service context (Sweeney and Soutar, 2001). This framework initially composed by three variables of value (functional, emotional, and social) was adapted to surf tourism by including the epistemic perspective (Williams and Soutar, 2009). Two value dimensions were added, experiential and contextual, based on current service marketing literature.

*Perceived value* is a multidimensional construct encompassing utilitarian (functional value, price) and socio-psychological (emotional, social, epistemic, experiential, and environmental values) aspects of consumption to explore surf camp consumer decision making (Sweeney and Soutar, 2001; see also more recently Kim and Thapa, 2018).

*Functional value* refers to service's ability to perform its purpose, either functional, utilitarian, or physical (Sánchez-Fernandéz and Iniesta-Bonillo, 2007). Common functional attributes include quality, durability, and price (Williams and Soutar, 2009). In the tourism industry, functional value relates to the quality of the resources influencing customers' experience (Jennings et al., 2009). Surf camp customers do place great importance on functional dimensions due to the inherent quality needed in adventure tourism operations because of safety issues and the level of planning needed to minimize risk. Therefore, we hypothesized the following:

*H1a - The functional dimension of the perceive service quality has a positive impact on surf camp customer's overall PV.*
*H1b - The functional dimension of the value for price has a positive impact on surf camp customer's overall PV.*

*Emotional value* refers to the service's ability to arouse feelings or affective states. Consumers evaluate the received service not only in terms of quality and performance, but also through internal feelings developed after consuming it (Al-Sabbahy et al., 2004). Customers usually develop their own emotional valuation of the whole act of the purchase and what they expect to receive (Sánchez et al., 2006). Emotional value is a key facet for consumers post-consumption value perceptions of adventure tourism experiences (Williams and Soutar, 2009). Value for individuals is phenomenologically determined by the beneficiary's perceptions at a specific point in time and location (Gummerus, 2013). For surfers, the quality of waves is a core aspect of the experience and it is connected with the socio-psychological dimension of value which is related to feelings, excitement, and exhilaration of the activity. Many surfers develop a heightened engagement that can nurture spiritual emotional benefits (Taylor, 2007). For our study, which is more concentrated on beginner and intermediate skilled surf tourists, the positive impact on the psychological dimension is believed to be strong but for experienced surfers, it had been established to be vast (Wiersma, 2014). Hence, it was hypothesized that:

*H1c – Emotional value of received services positively influences PV.*

*Social value* includes resources that can be linked to networks of members of a field defined as ‘the perceived utility acquired from an alternative's association with one or more specific social groups' (Williams and Soutar, 2009, p. 417). Social value is built around surfers' interactions with communities and their practices. Customers usually make an overall impression of the social value of the purchase by interacting with other surfers, the local communities, and the complementary shared moral beliefs of the local consumer. Social indicators may include social experience, other surf-related metrics such as surf community and surf events, clubs (board riders and lifesavers), history, public safety, and socio-psychological carrying capacity (Martin & Assenov, 2014). Thus, we hypothesized the following:

*H1d - Social value of received services positively influences PV.*

*Epistemic value* refers to the service's ability to ‘arouse curiosity, provide novelty, or satisfy a desire for knowledge’ (Williams and Soutar, 2009, p. 417). It is an important dimension in contexts where consumers are seeking new experiences, novelty and/or surprise (Sweeney and Soutar, 2001), such as tourism. Customers are simultaneously motivated by their socio–psychological desire to escape from their everyday routine of normal life and be stimulated by doing novel and adventurous things. Accordingly, we hypothesized the following:

*H1e - Epistemic value of received services positively influences PV.*

*Contextual (environmental) value* frames the resources and processes, and the overall service delivery (Chandler and Vargo, 2011). Tourism experience is likely to be influenced by the context the consumers are immersed in. The same is the case of surf camps: the contextual value is linked to an environmental dimension and the integration of natural resources (Horbel, 2013). Surfers care about, and value, environmental sustainability (Mach and Pointing, 2021), considering the conservation of the surf coast natural environment as a priority in the selection of surf destinations (Martin & Assenov, 2014). Thus, we hypothesized the following:

*H1f - Environmental value of received services positively influences PV.*

*Experiential value* is the self-oriented dimension of value in which a service value emerges from customers' appreciation of the service experience itself and is important for surfing as the activities and visual appeal offered to customers have a consequence on customers’ enjoyment of the experience (Mathwick et al., 2001). Surfing is the main experience, enhanced by the service design of the camps. Consequently, the following hypothesis was proposed:

*H1g - Experiential value of received services positively influences PV.*

### 2.5 Variables hierarchy and impact PV

Although all previous variables affect PV of surf camps, their effect on future behavior intentions, such as purchase, repeat, and recommend, neither act at the same time nor with the same intensity. Surfers tend to choose their surf destinations primarily based on the variety of waves and the quality of the natural environment (Barbieri and Sottomayor, 2013). Activities that enable tourists to pursue an embodied exploration of the self in empty wilderness settings encapsulate experiential value (Ponting and McDonald, 2013). Additionally, activities that offer experiences of protecting the natural environment —facilitating environmental value (namely sustainability)—may be an increasingly important but lower choice priority (Mach and Ponting 2021). Most surf tourists select their favored destination first before they search for a tour operator, their perspectives on sustainability or accommodation (Buckley, 2002). As a result, we hypothesized that:

*H1g - Surf camp customers choose the surf destination owing to natural attraction and considering surf services.*

*CS* refers to customers’ emotional state of mind of confirmation/disconfirmation of their perceptions about the service value, which is a comparison between the expectation of value (before the purchase) and the perceived post-purchase value (after the payment and consumption) (Sánchez et al., 2006). PV accumulates at various stages of the purchase process (Sweeney and Soutar, 2001) while CS is a post-purchase and post-consumption evaluation (Sánchez et al., 2006). PV not only is influential in the consumption phase but also affects CS, intention to recommend and return behavior in the post-purchase phase (Al-Sabbahy et al., 2004). It is necessary to measure the level of satisfaction to identify the value created by the service system (Williams and Soutar, 2009). Therefore, we hypothesized the following:

*H2 – PV positively influences CS.*

*Behavior loyalty* refers to customers’ behaviors including repurchase intentions, willingness-to-pay, and positive word-of-mouth communication (e.g., Zeithaml et al., 1996). As tourism follows an experiential nature, willingness to recommend previously experienced satisfactory services to others is an integral part of loyalty intentions, and surfers tend to show a strong disposition for surfing tourism (Barbieri and Sotomayor, 2013). Thus, we hypothesized that:

*H3 – CS will have a direct, positive, and significant association with intended loyalty behavior.*

**Figure 1:**
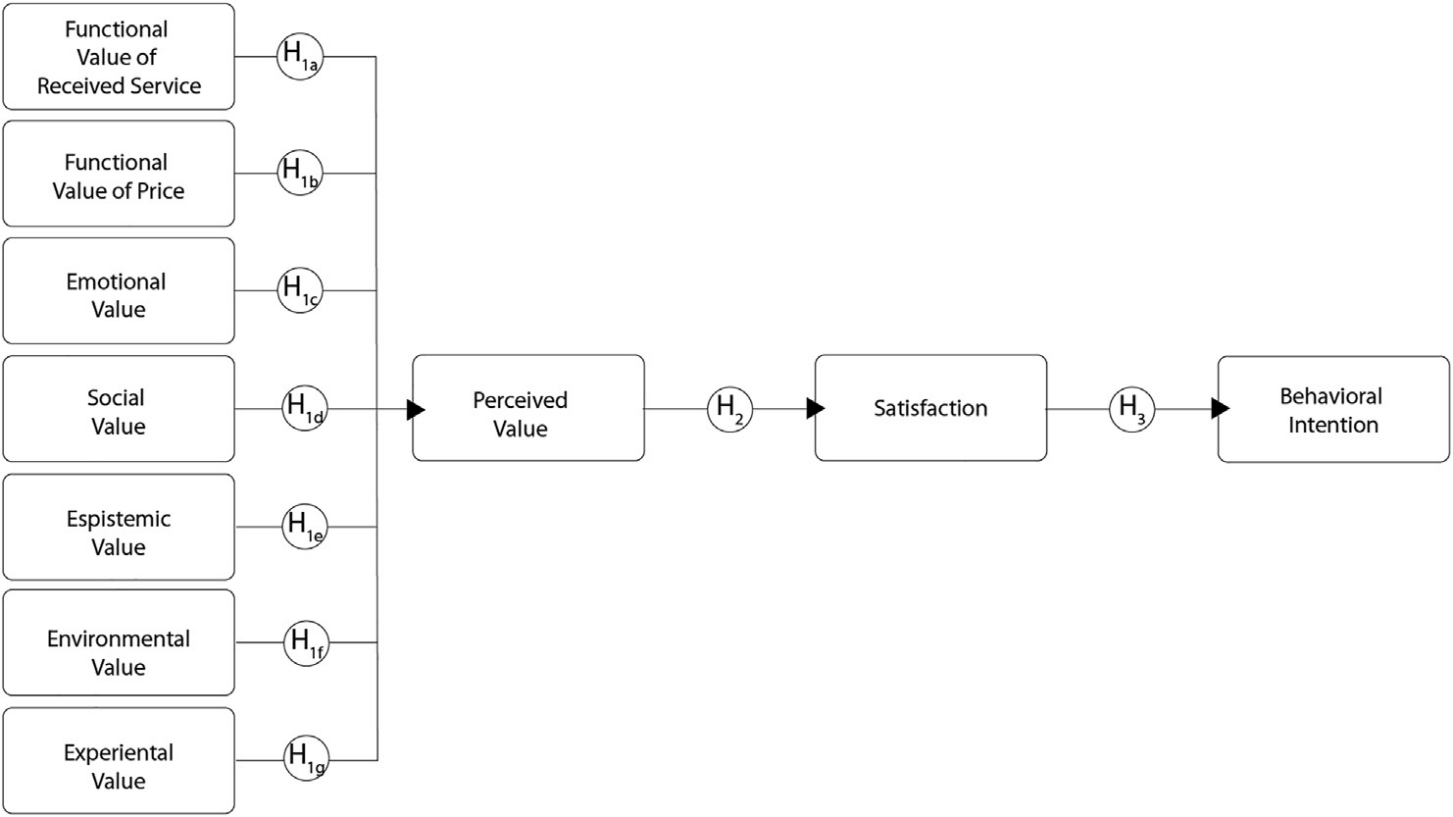
Conceptual model.

The conceptual model is presented in Figure 1.

## 3 Empirical study

### 3.1 Survey instrument

A questionnaire that included 52 questions was used to collect quantitative data from surf camp customers about PV, satisfaction, and future intentions. To ensure content validity, this study used previously validated instruments to measure the constructs in the proposed research model after appropriate adaptation to the context of this study (see Appendix 1). All items were measured using a 7-point Likert scale ranging from “1 = Strongly Disagree” to “7 = Strongly Agree” (Appendix 1). A pilot test was run on experts including academics, surfers, and surf camp customers to verify the internal consistency of questions.

### 3.2 Research context, data collection, and sample

Portugal is a world class surfing destination and a strategic location for competitive surfing events. The Portuguese coast has the morphology for surf practice with the country experiencing expansion of surfing-related businesses. Surfing is one of the activities included in the tourism strategy 2027 for Portugal developed by its national tourism organization (Turismo de Portugal, 2017). Portugal offers beaches (579), marinas, ports, and recreational docks (52) of recognized quality. One of the main businesses around these regions are surf camps, small hotels offering surf tours and classes, located mostly in tourist and popular surf spots. Young Western Europeans motivated by sport experience and natural environment factors visit these camps, which provide training and equipment.

**Figure 2:**
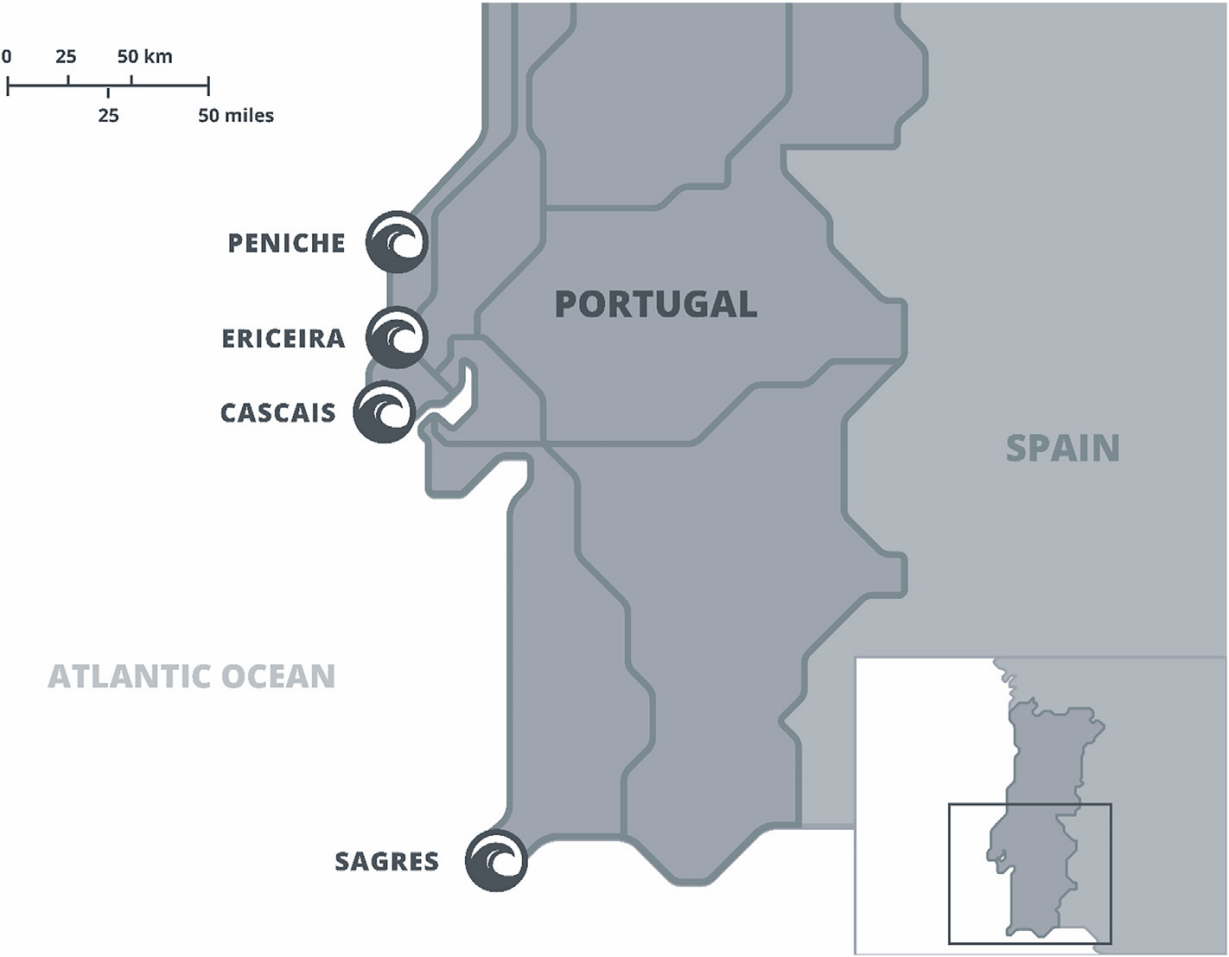
Map.

The model was tested with 300 tourists accommodated in surf camp establishments located in the leading surfing villages in Portugal, namely *Peniche, Ericeira, Cascais, and Sagres* (Figure 2). We used an internet search to get a fairly accurate overview of the surf camps existing in those villages and a list provided by the Portuguese Surfing Federation. Data were collected from surfers of these camps; they completed the online survey during a period of three months in the standard summer holiday, the high season for tourism in Portugal. A link to the survey was provided by flyers displayed around the camps and information displayed at reception desks. Surf camp accommodation businesses in Portugal are typically managed by passionate owner-operator who are also keen surfers themselves. The staff assisted greatly with survey link promotion and data collection. Within the camp, surfers often rest and converse between surfing sessions. Surfers wait for best prevailing conditions (e.g., tides, wind, or swell conditions) and have free time to participate. The communal nature of the camp coupled with higher levels of involvement of surfers in this recreation pursuit assisted data collection.

Respondents’ demographics, in terms of age and gender, were as follows: 75% were young adults between 21 and 35 years and 50.7% of participants were male. A clear majority of participants were from Northern European countries (72.1%) and most of the participants were employed (41.7%), followed by students (33.3%). These percentages suggest that the participants were visiting the surf camps for tourism. See Table 1 for the complete sample demographics.

**Table 1:** Respondent characteristics.

		Frequency	%
Gender	Male	152	50.7
	Female	148	49.3
Age	Under 21	57	19.0
	21–25	118	39.3
	26–30	72	24.0
	31–35	35	11.7
	Over 35	18	6.0
Nationality	German	74	24.7
	Swedish	52	17.3
	Norwegian	24	8.0
	Dutch	15	5.0
	Danish	14	4.7
	Swiss	14	4.7
	Austrian	12	4.0
	Finnish	11	3.7
	British	10	3.3
	Spanish	10	3.3
	Other Europeans	57	19.0
	Non-Europeans	7	2.3
Occupation	Employed	125	41.7
	Unemployed	1	0.3
	Student	100	33.3
	Unknown	74	24.7
Monthly Income	None	62	20.7
	Less than €1000	56	18.7
	€1000 to €1999	52	17.3
	€2000 to €3000	45	15.0
	More than €3000	42	14.0
	Unknown	43	14.3

### 3.3 Method

Previous research has recognized the potential of Structural Equation Modeling (SEM) in distinguishing measurement and structural models (e.g., Henseler et al., 2009). This study follows the variance-based technique, using Partial List Squares (PLS) for the following reasons: (a) the research model is considered complex; (b) the specific conceptual research model has not been tested in earlier literature; (c) not all items in our data are distributed normally (p < 0.01) based on a Kolmogorov-Smirnov test. For PLS estimation, the minimum sample size should satisfy one of the two following conditions: (i) ten times the largest number of formative indicators used to measure one construct or (ii) ten times the largest number of structural paths directed at a particular latent construct in the structural model (Hair et al., 2013). The sample comprised 300 respondents, implying that it met the necessary conditions for using PLS. Smart PLS M3 software was used to estimate the research model. The measurement model was first analyzed to assess reliability and validity, and then, the structural model was tested.

Assessing the results of formative measurement models requires a different approach to reflective measurement model evaluation as the indicators represent the construct's independent causes and they do not have to correlate highly (Hair et al., 2013). Using similar criteria associated with reflective measurement models including internal consistency reliability, convergent and discriminant validity is not appropriate and an adequate model fit cannot be expected (Wilcox et al., 2008). Instead, content validity, that is, ensuring that the formative indicators capture all relevant facets of the construct, based on a thorough literature review and theoretical grounding, is particularly important. Assessing formative measurement models includes the assessment of (1) convergent validity, (2) collinearity issues, and (3) significance and relevance of the formative indicators (Hair et al., 2013).

### 3.4 Results

Measurement models were estimated and checked with regard to validity and reliability. Construct reliability for the reflective measures ranged between 0.891 and 0.862 and the average variance extracted (AVE) ranged between 0.591 and 0.821. The assessment of the measurement models revealed that most loadings were acceptable within a range of 0.51 and 0.92 (PV: 0.51–0.86; CS: 0.88–0.92; and BI: 0.63–0.86).

Three common approaches were used to establish discriminant validity (Hair et al., 2013). First, the item-to-item correlation matrix demonstrated no visible issues. Second, all between construct correlations (Table 2) except those for “ID FBP” and “ID FBP Users” exceeded the accepted discriminant validity criterion of Fornell and Larcker (1981). Third, discriminant validity was corroborated with an inspection of the cross-loadings revealing suitable loading patterns. An inspection of the cross-loadings across the rows (Table 3) revealed that each item loaded higher on its respective construct than on any other construct. Inspection of the loadings down the column clearly illustrated that all items loaded highest next to their respective constructs.

**Table 2:** Measurement model.

Constructs	After No. of Ind.	Item Loading (λ) Range^a^	Alpha^b^ (α)	Comp Rel^c^ (ρ_ξX_)	AVE^d^
Functional value of Received Service (F)	7	-0.03–0.33	N/a	N/a	N/a
Functional value of Price (F)	3	0.10–0.70	N/a	N/a	N/a
Emotional Value (F)	3	0.32–0.47	N/a	N/a	N/a
Social Value (F)	4	0.13–0.39	N/a	N/a	N/a
Epistemic Value (F)	3	0.14–0.49	N/a	N/a	N/a
Environmental Value (F)	4	0.02–0.48	N/a	N/a	N/a
Experiential Value (F)	4	0.22–0.45	N/a	N/a	N/a
Perceived Value (F)	28	0.51–0.86	N/a	N/a	N/a
Satisfaction (R)	3	0.88–0.92	0.891	0.932	0.821
Behavioral Intention (R)	6	0.63–0.86	0.862	0.897	0.591

Note: a = Highest and Lowest Loading after Deletion; b = Cronbach's Alpha; c = Composite Reliability; d = Average Variance Extracted (AVE). Weights reported in the loading range column when it is a Formative (F) construct.

**Table 3:** Correlation between constructs and AVE.

	1	2	3	4	5	6	7	8	9	10
1. Behavioral Intention	*0.770*									
2. Emotional Value	0.613	*1.000*								
3. Environmental Value	0.468	0.497	*1.000*							
4. Epistemic Value	0.515	0.512	0.572	*1.000*						
5. Experiential Value	0.583	0.613	0.582	0.620	*1.000*					
6. Functional Value of Price	0.559	0.612	0.510	0.483	0.567	*1.000*				
7. Functional Value of Received Service	0.589	0.670	0.468	0.451	0.562	0.627	*1.000*			
8. Perceived Value	0.684	0.861	0.666	0.674	0.760	0.636	0.718	*1.000*		
9. Satisfaction	0.753	0.680	0.561	0.572	0.647	0.554	0.610	0.745	*0.906*	
10. Social Value	0.527	0.623	0.578	0.597	0.608	0.521	0.516	0.671	0.579	*1.000*

Note: Square Root of AVE on diagonal is presented for Reflective (R) Constructs only.

Diagonal elements (in italics) are AVE of each construct.

### 3.5 Analysis of structural relations and hypothesis testing

**Figure 3:**
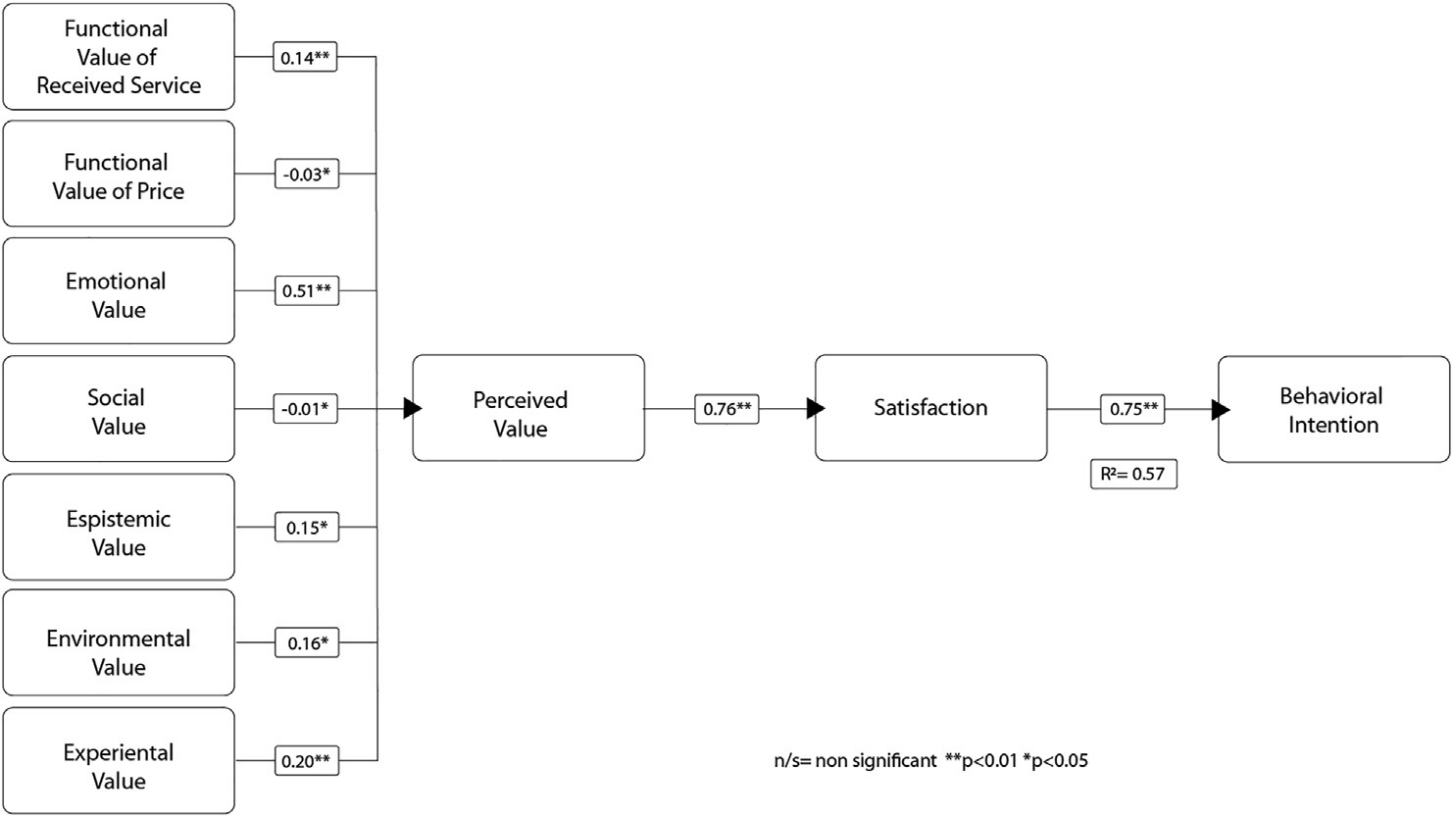
Structural model results.

Table 4 presents the results of the structural model with significant effects demonstrated for the relationships. The predictive capacity of this model was strong with an R^2^ = 0.57 (Figure 3).

**Table 4:** Structural model results.

Structural Relation	Model 1 (Main Effects)		
	Path Coeff	Sig	f^2^
Functional Value of Received Service → Perceived Value	0.14	∗∗	0.08
Functional Value of Price → Perceived Value	-0.03	∗	0.00
Emotional Value → Perceived Value	0.51	∗∗	0.87
Social Value → Perceived Value	-0.01	∗	0.00
Epistemic Value → Perceived Value	0.15	∗∗	0.10
Environmental Value → Perceived Value	0.16	∗∗	0.12
Experiental Value →Perceived Value	0.20	∗∗	0.15
Perceived Value → Satisfaction	0.76	∗∗	1.25
Satisfaction → Behavioral Intention	0.75	∗∗	1.31
*R*^*2*^		0.57	

Note: Bootstrapping results (n = 5.000) ∗∗*p* < 0.01 ∗*p* < 0.05.

Path Coeff = Path coefficient; Sig = Significance; f^2^ = effect size.

Emotional value exercises the strongest influence on PV (0.508). This is followed by experiential value (0.204), environmental value (0.164), epistemic value (0.152), and functional value of services (0.144). The result of social value (-0.013) is somewhat counterintuitive since experiences involving services shared are often expected to be driven by social values (Sheth et al., 1991). This premise will be explored further in the discussion. Results show that although functional value of price has a negative impact, it is rather small (-0.031).

Finally, PV exerts a strong influence on CS (0.745) and CS impacts BI (0.753). We now discuss the results.

## 4 Discussion

Our research studies consumer perceptions of value derived by the participants of surf camps. The key constructs included in the analysis include relevant facets of PV, CS, and BI. This latent variable combination proved to be the best surrogate to represent the buying process, service evaluation, recommendation, and other stages that are strongly interconnected. PV is an important inclusion to assess the complex phenomena that fully represents the consumers’ perception of value and is considered integral in driving satisfaction (Cronin et al., 2000). PV is a predictor of BI to repurchase and recommend confirming the prior contributions of Williams and Soutar (2009). Consequently, PV is not only influential at the final purchase phase but also impacts satisfaction and the intention to recommend and engage in return behavior patronage in the post-purchase phase (Al-Sabbahy et al., 2004). Our results demonstrate the respective influence of drivers that determine PV.

### 4.1 Research contributions

Considering that the surf camp industry is not widely studied within the tourism field, this study provides some further insight into surfer conduct and the respective driving forces affecting experience. Delineating by the types of value also represents an advancement. This study identifies the different factors that determine the PV of the total surf camp experience. This is the most valuable contribution and is complementary of work demonstrating the impact of value on satisfaction, intention to recommend, and return behavior in the post-purchase phase (Al-Sabbahy et al., 2004).

Variables such as emotional value, experiential value, functional value of received service, and functional value of price are key constituents of overall PV. Emotional value showed the strongest influence and the largest weights were presented by the feelings of wellbeing generated by the surf camp experience and the extent to which the customer felt appreciated by staff. Emotional value stands as a social-psychological dimension that depends on a product's ability to arouse feelings or affective states, and emotional responses are likely to occur in adventure tourism experiences, hence complementing the surfing experience (Williams and Soutar, 2009). Such measurement variables have a positive correlation, and a stronger perception will result in higher PV. The second latent variable was experiential value and the largest item weights were the measurement variables associated with free time. Experiential value had a strong impact very much in line with the activity's role in creating enriching tourists' experiences. Owing to the experiential nature of tourism services, experiential factors have been incorporated (Otto and Ritchie, 1996). The experiential view has been favored over the information processing approach. Leisure activities, like tourism, need to resort to fantasies and feelings (intangible attributes) to explain purchasing behavior (Scott et al., 2009). The third strongest influence was presented by the functional value for the received service with the main weights being the add-on services and the respective facilities of the camp. Epistemic value and environmental value contribute positively toward PV but to a lesser extent than the previously mentioned constructs. Innovation and exploration of new environments with surfing and camp locales are considered helpful for operators to satisfy the desires of adventure tourists. Surfers have an appetite for unique and novel experiences while on holiday (Weber, 2001). Epistemic value is considered a key component of the adventure tourism experience since it includes the novelty of the activity and the destination. This is a key factor in many adventure tourism products (such as surf camps) owing to the tourists' desire for exploratory, novelty seeking and variety seeking behavior (Zuckerman, 1994). In adventure tourism, functional value is important because of safety issues and the planning needed to minimize risk (Williams and Soutar, 2009).

Moreover, the functional value of price and social value latent variables revealed a negative effect impacting PV. Studies on social values highlight that surfers do not like crowds and travel in small groups of less than four people to escape them (Dolnicar and Fluker 2003; Barberi and Sotomayor 2013). The main weight for the functional value of price was the measurements associated with adequate return for money, representing a sound purchase for the price paid whereas the main weight for the social value was the measurements associated with the way the customer is perceived and the interactions of the tourist with other tourists, locals and residents. The functional value of price is a small negative coefficient, which is not surprising since a wide majority of surf camp customers come from abroad, mainly from Northern European countries. For them, the price variable is not at the forefront of their decision-making process. Functional value of price (value for money) is associated with the part of PV that is related to sacrifices made by customers. In value models, quality and price have been treated as separate influences on PV with quality having a positive influence, whereas price has a negative influence (Doods et al., 1991). This result could be explained by the low-price elasticity behavior of customers and challenges the common idea that services shared with others is often driven by social values (Sheth et al., 1991). Surfing, in some ways, is an anti-group activity and there is an inflection point where the prevailing crowd-level negatively impacts the experience. This is also a consideration for surf safety given the surfer's experience levels against prevailing surf break conditions. This point merits future exploration.

Finally, the results illustrate the predictive capacity of the model (R^2^ = 0.57). This result supports the adopted structural model approach and necessitates the inclusion of PV in tourism studies in the future. It is clear that the impact of PV on satisfaction and subsequently, for satisfaction impacting BI is quite strong. Tourism researchers have begun to address the need to incorporate a multidimensional value perspective and have examined its relationship with other post-consumption constructs, such as CS and BI (Petrick, 2002).

In a post COVID-19 environment, we anticipate that building BI is crucial for surf camps since it represents the extent to which the customer will recommend the experience perhaps in a business environment where prevailing surf camp prices might rise (i.e., camps trying to recoup recent losses, meet stringent testing or regulatory demands or to meet heightened customer expectations regarding delayed gratification and altered communal eating or more stringent cleaning requirements). The social dynamics will be different. Therefore, this factor could affect the demand for the service and impact economic benefits.

### 4.2 Managerial contributions

As discussed, a strong relation could be observed between PV and BI. Thus, the main managerial contributions should aim to increase the level of PV since it could lead to an increase in the demand for the service.

The first set of recommendations should aim to increase the general wellbeing of the customer and the degree to which the customer feels appreciated by staff. To achieve such a goal, staff should be well trained in customer service and establish a customer-centric orientation. Customers usually develop their own emotional evaluation of the complete act of the purchase and what they expect to receive (Sánchez et al., 2006). This means that surf camps should aim to present and create realistic expectations to their customers, to avoid affecting the emotional value perceived.

Furthermore, market segmentation should be performed to target specific parts of the population that may have significant knowledge on sustainable development and environmental protection, such as nature-based adventure surfers (Orams and Towner, 2012). Building on Cheng and Wu's (2015) findings: targeting sustainable savvy consumers, along with an effective marketing strategy that communicates sustainable surfing experiences, could result in an efficient combination to significantly increase PV. In the post COVID-19 travel and camp landscape, socially responsible surf camp operators will be sensitive to the prevailing personal distancing measures, testing, vaccination checks, and adopting a broader responsibility for more stakeholders including staff, surfers, and the community at large. An unexpected benefit of the pandemic will see an increased awareness respecting local cultures, greater environmental protection, and widespread adoption of sustainable tourism practices. Mach and Ponting (2021) suggest that surf tourism may dramatically transform with surfers paying a premium to nurture sustainability.

Fundamentally, surf camps should aim to maximize the enjoyment of free time. Experiential value is particularly important in the tourism industry as the activities and visual appeal, driven by the service design, offered to customers have a positive effect on customers’ enjoyment of the experience (Mathwick et al., 2001). Staff should also aim to promote the enjoyment of free time with mixed activities. Surf camps should include add-on services and facilities that meet the basic requirements (e.g., reception area, kitchen, rooms, and common areas). This is core to building community relations for camp alumni advocates. Some other add-on services that could be considered by the owner could be implemented, such as yoga programs and the elaboration of handcrafts (e.g., necklaces, bracelets, and accessories). These may be used as complementary activities to surfing, which could be key for tourists interested in surfing, but enhance the overall experience. Finally, surf camps should aim to reinforce the surf identity of the community and tourists, since this element presented the highest weight of the environmental value construct, which positively affects the level of PV.

Furthermore, all these activities and add-on services should be properly tailored to the target segments (see, for example, Orams and Towner, 2012). There are certain key demographic differences between surfers that could help determine the appropriate services that establishments could provide. Porter and Usher (2019) suggest that older, married, or parent surf users are willing to pay more for services. Others use the surfing experience as a means of acquiring time for themselves away from family or partners, preferring friends as travel companions. All these factors should be taken into consideration when offering additional activities and establishing the marketing strategy.

Additionally, a primary attraction of the surf camps is undoubtedly the diverse ecosystems. Thus, ecosystem preservation should definitely be taken into consideration in the business operation since this affects holistic PV. Surf camp business models should not only be concerned with maximizing profit but also about preserving nature and minimizing the carbon footprint (Mach and Ponting, 2021). Coastal developments and surf camp expansions can affect the prevailing water quality, sometimes creating a polluted, unhealthy environment to surf in or even affecting wave characteristics (Bicudo and Horta, 2009). Yet, most surfers develop a strong sense of attachment to these places (their breaks). There is an implied code that facilitates sustainability and protective codes.

Moreover, considering the measurement variable “good return for money,” it is believed that this could be understood as the price/quality proposition, giving a stronger emphasis to the functional value of price. Conversely, there was a negative correlation among the functional value of price and PV. This is a notable insight since the surf camps service demonstrated probable low-price elasticity, meaning that the general PV of the experience is not determined by price. Managers should consider this information while setting the price since a small increase in rates (that could lead to an increase in profit) would not greatly affect the resultant BI of customers.

Finally, the latent variable of social values shows that customers tend to consider surf camps and individual journeys. The fact that this construct has a negative relation with PV illustrates that individuals value their own privacy or individual pursuit. This is a major contribution since an excess of integration activities and group tasks could harm the experience of such surfers. It is recommended that surf camps should ideally design some spaces for inner contact and in some way, design break out spaces that limit the interactions among customers.

### 4.3 Study limitations and future research directions

Future studies could complement the results and findings exhibited in this paper. One would be to further study the price elasticity of the respective surf camps and regions, for managers to understand the impact of price variations upon the total demand of the service. Another important contribution could be to study which elements drive the expectations of different segments for a surf camp. This study found that achieving or exceeding these expectations greatly affects PV of the experience, which is why it is important to understand the rational steps by which the customer builds such expectations.

Although this study contributes to solving the dearth of literature on value creation in surf camps, it does have some restrictions that are worth mentioning. This study was conducted solely in Portugal and other well-known surf camps located in other countries such as Indonesia, Costa Rica, and Panama were left out. For a better representation of surfer behavior, a more comprehensive study should involve different geographical locations for data gathering. This study represents mostly European respondents and behaviors, and it should be considered that the recommendations and conclusions would not be suitable for a drastically different context such as Latin American. It is recommended to study the difference among surf camps in different geographical locations. This would enable a deeper understanding of the competitive advantage expected by one camp location, and their add-on services, among other differentiating factors offered by each one. This would lead to a better understanding of the most appropriate business model and could conclude with significant managerial contributions based on exemplar cases.

## Declarations

### Author contribution statement

Paulo Rita, Brad Wilson, Andres Barrios and Bastian Popp: Conceived and designed the experiments; Performed the experiments; Analyzed and interpreted the data; Contributed reagents, materials, analysis tools or data; Wrote the paper.

### Funding statement

This research did not receive any specific grant from funding agencies in the public, commercial, or not-for-profit sectors.

### Data availability statement

The data that has been used is confidential.

### Declaration of interests statement

The authors declare no conflict of interest.

### Additional information

No additional information is available for this paper.

## Appendix A Supplementary data

Appendix_SupplementaryFile_Accepted_Final Appendix_SupplementaryFile_Accepted_Final
