# Supplementary material for: Extending the notion of customer value to surfing camps
Source: Heliyon. 2021 Aug 26;7(8):e07876. doi: 10.1016/j.heliyon.2021.e07876 (PMC8403551; doi:10.1016/j.heliyon.2021.e07876)
Supplement: Appendix_SupplementaryFile_Accepted_Final [file mmc1.docx]

# Appendix 1

| **Functional value of the received service** *(Sweeney and Soutar (2001) Williams and Soutar (2009); Baum and Chan (2007); Cronin et al. (2000); Gallarza and Saura (2006); Sanchéz et al. (2006)* | |
| --- | --- |
| fvrc_1 | The surf camp has been providing a consistent quality service |
| fvrc_2 | The surf camp facilities meet my requirements (reception area; kitchen; rooms; common areas, cleanness) |
| fvrc_3 | The surf camp had competent employees and accredited surf teachers (knowledgeable and skillful) |
| fvrc_4 | The surf equipment that has been provided is in good conditions |
| fvrc_5 | The surf camp is neat and clean |
| fvrc_6 | The surf camp has been providing a well-organized service |
| fvrc_7 | The surf camp provides add-on services that I was looking for (alternative programs and activities; sense of place; bar/restaurant) |
| **Emotional value of the received service** *(Sweeney and Soutar (2001) Williams and Soutar (2009); Baum and Chan (2007); Cronin et al. (2000); Gallarza and Saura (2006); Sanchéz et al. (2006)* | |
| evrc_1 | The surf camp gave me feelings of well being |
| evrc_2 | I felt really appreciated by the staff |
| evrc_3 | With the surf camp service, I have been getting the feeling of surf triumph |
| **Social value of the received service** *(Sweeney and Soutar (2001); Williams and Soutar (2009); Gallarza and Saura (2006); Sánchez et al. (2006))* | |
| svrc_1 | This surf camp experience would improve the way I am perceived |
| svrc_2 | The surf camp has been promoting interaction and relationships among the other tourists inside the surf camp |
| svrc_3 | The surf camp experience has promoted contact and interaction with locals and residents |
| svrc_4 | The surf camp has reinforced the feeling of belonging to a group |
| **Experiential value of the received service** *(Mathwick et al (2001); Gallarza and Saura (2006))* | |
| evrc_1 | The surf camp decoration and design has been offered me pleasure |
| evrc_2 | The beauty of landscapes and surf camp location have been contributing for my fulfillment |
| evrc_3 | The surf camp has been providing me absorbing activities |
| evrc_4 | The surf camp has been promoting the enjoyment of my free time |
| **Environmental value of the received service** *(Baum and Chan (2007); Assanov and Martin (2014))* | |
| envrs_1 | The surf camp has been promoting the local community spirit |
| envrs_2 | The surf camp reinforces the surf identity of the community and tourists |
| envrs_3 | The surf camp promotes sustainable and conservative practices of the environment (fauna and flora; coastal erosion; waves degradation) |
| envrs_4 | This surf camp promotes and protect the quality of waves |
| **Functional value of the price** (Sweeney and Soutar (2001); Soutar and Williams (2009)) | |
| fvp_1 | The surf camp was reasonably priced |
| fvp_2 | It was a good purchase for the price paid |
| fvp_3 | It was a good return for money |
| **Epistemic value of the received service** (Williams and Soutar (2009); Baum and Chan (2007)) | |
| epvrs_1 | The experience has been made me feel adventurous |
| epvrs_2 | The experience has been satisfying my curiosity |
| epvrs_3 | The surf camp has been arousing the feeling of escaping |
| **Satisfaction** (Sweeney and Soutar (2001); Williams and Soutar (2009) ) | |
| s_1 | The experience has satisfied my needs and wants |
| s_2 | It was a good experience |
| s_3 | My choice to purchase this surf camp was a wise one |
| **Behavioral Intentions** (Sweeney and Soutar (2001); Williams and Soutar (2009)) | |
| bi_1 | I would recommend this surf camp experience to friends and relatives |
| bi_2 | I would recommend this surf camp experience through electronic social networks |
| bi_3 | I would go on other surf experiences in future |
| bi_4 | The surf camp encourages me to surf again |
| bi_5 | I would go to a surf camp again |
| bi_6 | I am willing to take the same surf camp accommodation |
